# A 22-Year Survey of Leishmaniasis Cases in a Tertiary-Care Hospital in an Endemic Setting

**DOI:** 10.3390/ijerph110302834

**Published:** 2014-03-10

**Authors:** Adriana Calderaro, Sara Montecchini, Sabina Rossi, Chiara Gorrini, Maria Loretana Dell’Anna, Giovanna Piccolo, Maria Cristina Medici, Maria Cristina Arcangeletti, Carlo Chezzi, Flora De Conto

**Affiliations:** Unit of Microbiology and Virology, Department of Clinical and Experimental Medicine, Faculty of Medicine and Surgery, University of Parma, Viale A. Gramsci 14, 43126 Parma, Italy; E-Mails: sara.montecchini@unipr.it (S.M.); srossi@ao.pr.it (S.R.); cgorrini@ao.pr.it (C.G.); mdellanna@ao.pr.it (M.L.D.); giovanna.piccolo@unipr.it (G.P.); mariacristina.medici@unipr.it (M.C.M.); mariacristina.arcangeletti@unipr.it (M.C.A.); carlo.chezzi@unipr.it (C.C.); flora.deconto@unipr.it (F.D.C.)

**Keywords:** visceral leishmaniasis, cutaneous leishmaniasis, *Leishmania* spp., diagnosis, culture, epidemiology

## Abstract

The northward spread of leishmaniasis from Mediterranean to Continental Europe affects our area where it is typically associated with *Leishmania infantum* infection. In this study a 22-year survey was performed in patients (including both patients with and without history of travel through endemic areas other than Italy) attending the University Hospital of Parma, Northern Italy, in order to make a contribution to describe the cases of the visceral leishmaniasis (VL) and cutaneous leishmaniasis (CL) diagnosed in this area. One hundred fifty-six samples from 134 patients with clinical suspicion of leishmaniasis (96 suspected of having VL, 37 CL and one both VL and CL) were analyzed in our laboratory during 1992–2013 by microscopy, culture and, from 2005, also by real-time PCR. *Leishmania* spp. were detected in 23 samples of 15 patients (seven with VL and eight with CL), representing an infection rate of 11.2%. The figure of the cases of leishmaniasis herein reported, even if not comparable to that described for Italian areas other than Parma, underlines that suitable tools are mandatory for correct diagnosis. Moreover, the severity of this disease, particularly VL with its documented northward spread, requires physicians of continental Europe to increase their attention about the possibility of suspecting leishmaniasis in patients reporting related signs and symptoms and/or risk factors.

## 1. Background

Leishmaniasis is a parasitic infection caused by more than 20 species of protozoa belonging to the genus *Leishmania* transmitted to mammals (including human beings) by phlebotomine sandflies [1,2], and infected dogs represent the main domestic reservoir host [2]. It is associated with three main types of disease manifestations: visceral, cutaneous, and mucocutaneous [1].

As determined during the Sixtieth World Health Assembly there are 98 countries and three territories where *Leishmania* is endemic (350 million people at risk), with the majority of cases occurring in developing countries, resulting in approximately 500,000 cases of visceral leishmaniasis (VL) and 1.5 million cases of cutaneous leishmaniasis (CL) each year and an estimated 50,000 deaths annually [3]. 

It is noteworthy that human immunodeficiency virus (HIV) and *Leishmania* reinforce each other in a detrimental manner [3]. Visceral leishmaniasis is more likely to develop in HIV-infected patients and impairs their response to antiretroviral treatment [3]. The global spread of HIV infection was responsible, in fact, starting from the mid-1980s, for the upsurge of HIV/VL coinfection in four Mediterranean countries (*i.e.*, Spain, France, Italy, and Portugal), with such cases occurring almost exclusively among intravenous drug users [1].

In Europe, both CL and VL are well established diseases in the Mediterranean basin, with an incidence ranging from 0.02/100,000 to 0.49/100,000 that roughly translates to 700 cases per year [1], but in the recent years a northward VL spread from endemic Mediterranean zone to Continental Europe was observed [1,4,5].

In Italy autochthonous leishmaniasis appears not to be limited to the Mediterranean region anymore [1,6,7], providing in 2003–2005 the first evidence in Europe of the emergence and northward spreading of VL transmission as a probable result of climatic modifications, associated to global warming (influencing dispersion and density of sandflies), and human behavioral factors (importing infected dogs into non-endemic areas) [8].

In Italy, zoonotic VL is considered endemic, and sporadically zoonotic CL occurs, both caused by *L. infantum* [8]. It was estimated that 450–500 cases occurred both in 2004 and 2005 taking into account, however, that only the cases that are diagnosed and treated in hospitals are reported, while those diagnosed in private clinics are not [8].

Our laboratory is part of a 1,218-bed tertiary-care University Hospital located in a continental region of Italy where recently an outbreak of leishmaniasis was reported [9]. The aim of this study was to describe the cases of leishmaniasis diagnosed in our area during a 22-year period (1992–2013), using the data obtained by the application of the routinely diagnostic practice, including cultivation and PCR. 

## 2. Methods

### 2.1. Study Area and Population

The study was conducted at the University Hospital of Parma, a 1,218-bed tertiary care centre (year 2012) with over 50,000 admissions per year registered in the study period [10]. The province of Parma, located in the Northern Italy, has 445,283 inhabitants [11]; the population attending this hospital was estimated in 207,594 inhabitants, 10% of whom were immigrants from developing countries [12].

### 2.2. Samples and Patients

One hundred-ten samples belonging to 96 patients with the clinical suspicion of VL, 44 samples belonging to 37 patients with the clinical suspicion of CL, and two samples belonging to one patient with the clinical suspicion of both VL and CL, for a total of 156 samples from 134 patients, all reporting signs and symptoms and/or risk factors related to leishmaniasis, were analyzed in our laboratory during the period 1992–2013. The samples analyzed in this study are reported in Table 1.

**Table 1:** Samples analyzed in this study.

Patients	Samples	
	Number	Type
88	96	Bone marrow
29	31	Skin biopsy
5	5	Skin lesion aspirate
2	2	Lymph node aspirate
1	2	Peripheral blood
1	1	Lymph node biopsy
3	3	Skin lesion aspirate
	5	Skin biopsy
2	2	Bone marrow
	2	Lymph node biopsy
1	2	Bone marrow
	1	Peripheral blood
1	1	Bone marrow
	1	Spleen biopsy
1	1	Bone marrow
	1	Skin biopsy
134	156	Total

On the basis of the available information, patients whose samples were analyzed for CL all presented with ulcerative skin lesions consistent with the suspicion of leishmaniasis: in two cases the lesions were accompanied by itching. The principal reported signs and symptoms of patients with the clinical suspicion of VL were the following: bone marrow aplasia, fever, hepatosplenomegaly, anemia, lymphoadenopahty, thrombocytopenia, lymphadenopathy, leukopenia, arthralgia, ipergammaglobulinemia, and asthenia. In one case weight loss and sweating were also reported, and in one case neutropenia was present.

One hundred-one patients out of the 134 were Italians and 33 immigrants from developing countries, 103 were adults, 31 children, 83 were male and 51 were female. Clinical data about patients revealed three HIV-infected patients (two suspected of having VL and one suspected of having CL), one patient with AIDS suspected of having both VL and CL, one immunocompromised due to unknown causes, one immunosuppressed patient and three transplant patients suspected of having VL. Among the Italian patients, one suspected of having CL reported travels (humanitarian mission) through Africa, one suspected of having CL reported travel to Brazil, one suspected of having VL reported travel to the Far East, and one suspected of having VL reported a number of trips to the island of Crete in the previous two months. 

### 2.3. Conventional Parasitologic Assays

#### 2.3.1. Microscopic Examination

Sixty-five bone marrow samples, two skin lesion aspirate samples, two blood samples and two lymph node aspirate samples were immediately used to prepare four thin films each: two stained with acridine orange and two with 1% Giemsa in phosphate-buffered saline (pH 7.0), respectively, as already described [13] for microscopic observation. Two thick films also were stained with 1% Giemsa in phosphate-buffered saline (pH 7.0) for microscopic observation. Nineteen skin biopsies, two lymph node biopsies, and one spleen biopsy were each used to prepare an imprint smear subsequently stained with Giemsa, according to standard procedures [14].

#### 2.3.2. Cultivation of *Leishmania* Promastigotes

One-hundred-two bone marrow samples, 33 skin biopsies, five skin lesion aspirate samples, one spleen biopsy, two lymph node aspirate samples, three lymph node biopsies, and three blood samples were cultivated by using two different slant agar media: Evan’s modified Tobies’s medium and Novy Nicolle-MacNeal (NNN) medium with or without antibiotics. Evan’s modified Tobies’s medium was prepared according to standard procedures [15] with some modifications. Briefly, after the preparation of the mixture in sterile double-distilled water (100 mL), 17 mL of defibrinated horse blood (Biolife Italiana srl, Milano, Italy), with or without penicillin G (2,000 U.I.) (Sigma-Aldrich Corporation, St. Louis, MO, USA) and streptomycin (20,000 U.I.) (Bristol-Myers Squibb, Latina, Italy), were added to 50 mL of the solution, and then 1.5 mL of the obtained medium were dispensed in screw-cap culture tubes. NNN medium was prepared according to standard procedures [16] with some modifications. Briefly, 2,000 U.I. of penicillin and 20,000 U.I. of streptomycin were added to 10 mL of the defibrinated rabbit blood (Sclavo Diagnostics International, Siena, Italy—until 2012; Istituto Zooprofilattico della Lombardia e dell’Emilia-Romagna, Brescia, Italy—from 2013), and then 1.5 mL of the obtained medium were dispensed in screw-cap culture tubes. Before the inoculation 0.2 mL of overlay solution, prepared as previously described to be used with Tobie’s medium [15], were added to each of the tubes containing the different media. When available, 1, 2, 3, 4 drops of all the liquid samples and the biopsies samples broken with a mortar in the overlay solution, were each added to the tubes. The *Leishmania* promastigotes were cultivated at 25 °C for 4 weeks: During the first 2 weeks the cultures were examined 2 times per week, for the latest 2 weeks one time per week. A subcultivation every 7 days in fresh medium was performed from the tubes inoculated with 3 or 4 drops of sample.

### 2.4. Molecular Methods

#### 2.4.1. Isoenzyme Characterization

Nine out of the positive samples for the presence of *Leishmania* spp. by microscopy and/or culture from 1992 to 2001 were submitted to isoenzymes characterization at the Istituto Superiore di Sanità in Rome, as previously described [17].

#### 2.4.2. FRET Real-time PCR Assay

DNA was extracted from 200 μL of 36 bone marrow samples, four skin lesion aspirate samples and from 25–30 mg of 17 skin biopsies and one splenic biopsy, belonging to a total of 52 patients by using the High Pure PCR Template Preparation kit (Roche Diagnostics, Mannheim, Germany), according to the manufacturer’s instructions [13]. The extracted DNA was used immediately for PCR assays or frozen at −20 °C until analyzed. A Fluorescence Resonance Energy Transfer (FRET) Real-time PCR targeting a portion of the 18S rDNA of *Leishmania* spp. [18] was used with minor modifications. Briefly, the 20 μL reaction mixture contained the following: 4 μL of 5× Light Cycler FastStartDNA MasterPLUS Hybridization Probes [a component of the Light Cycler FastStartDNA MasterPLUS Hybridization Probes kit (Roche Diagnostics) in which the concentrations of MgCl_2_ and inhibitor-binding proteins are optimised], 350 nM primer CDLS, 500 nM primer CDLA (Eurogentec, Liege, Belgium), 200 nM probe CDLP5, 200 nM probe CDLP3 (TIB Molbiol S.R.L., Genova, Italy) and 8 μL of DNA. Real-time FRET PCR was performed in glass capillary tubes on a 2.0 Light Cycler instrument (Roche Diagnostics). Reaction conditions were: Initial denaturation at 95 °C for 15 min, followed by 45 cycles of 95 °C for 10 seconds, 55 °C for 10 seconds, and 72 °C for 40 seconds. Readout was performed in the channel F2/Back F1. A sample was regarded as positive when the Light Cycler software determined a crossing point in the analysis screen. As a negative control for specimen processing, DNA was extracted from a sample that did not contain the target sequence (sterile double-distilled water) and the extract was subjected to amplification and detection. As a negative control of amplification, reaction mixture without DNA was amplified; as a positive control, from 2005 to 2011 the DNA from a reference *L. major* strain was used and from 2012 till now *L. infantum* purified DNA (Vircell S.L., Granada, Spain) was used. 

The absence of PCR reaction inhibitors in the analyzed samples was assessed by a Taq-Man based Real-time PCR assay specific for human β-actin DNA (Human ACTB Endogenous Control VIC/TAMRA Probe, Primer Limited; Applied Biosystems, Foster city, CA, USA), as previously reported [13]. The FRET Real-time PCR was introduced in the laboratory diagnosis on the end of 2005 and since then all samples were also tested with this assay (37 for the diagnosis of VL and six for the diagnosis of CL). 

## 3. Results

On the total of the 156 analyzed samples belonging to 134 patients, *Leishmania* spp. were detected in 23 samples belonging to 15 patients, corresponding to an infection rate of 11.2%; VL was diagnosed in seven patients (10 samples) and CL was diagnosed in eight patients (13 samples). The demographic, epidemiological and clinical data of the patients with leishmaniasis are reported in Table 2.

The infection rate of leishmaniasis with regard to the travel history of the patients was 21.62% (8/37) in immigrant patients and in Italian patients with documented travel through endemic areas and 7.21% (7/97) in Italian patients without history of travel through endemic areas, with regard to age was 13.59% (14/103) in adult patients and 3.22% (1/31) in pediatric patients, with regard to sex was 14.46% (12/83) in male patients and 5.9% (3/51) in female patients. 

On the basis of the available data, significant differences were not revealed in the sets of clinical signs and symptoms associated with patients with leishmaniasis *versus* patients not infected with *Leishmania* spp., both for VL and CL. None of the HIV-infected patients included in this study revealed an infection by *Leishmania* spp.

The isoenzymes characterization revealed the presence of *L. major* zymodemes MON 25 in two skin biopsies, *L. major* zymodemes MON 196 in one skin biopsy and *L. infantum* zymodemes MON 1 in six bone marrow samples, as reported in Table 2. 

A comparison of the results of microscopic examination, culture and real-time PCR performed on the samples belonging to the patients (crossing point values ranged 29.07 to 34.80 cycles) with leishmaniasis diagnosed in this study is reported in Table 3.

## 4. Discussion

As recommended by the World Health Organization (WHO) a concerted research effort should be made to define the population at risk and the global burden of leishmaniasis accurately, being several aspects of the epidemiology and transmission of this disease unknown and requiring research [3].

In Italy, where the total population is 60,483,521 inhabitants with 32% living in rural areas, zoonotic VL is endemic, and sporadically zoonotic CL occurs, both caused by *L. infantum*, especially in the Mediterranean basin [4,8]. However, a northward VL spread from the endemic Mediterranean area to continental Europe involved also our country maybe due to a 30-year expansion of its vectors, belonging to the genus *Phlebotomus*, towards northern latitudes associated with current global warming [3,4]. The vectors involved in the transmission of the disease evidently increased in density and expanded their geographic range in northern continental Italy [4]. From 1990 through early 2005, 230 VL cases among residents in northern Italy occurred, representing 10.9% of all Italian cases (2,139) reported in the same period. It was in fact postulated that continental northern Italy is now focally endemic for VL and that a moderate risk for human disease does exist, although the current intensity of *Leishmania* transmission seems to be lower than in traditional settings of Mediterranean VL [4].

Interestingly, a strikingly high seroinfection rate (7.4%) of *Leishmania* specific antibodies detected by western blot was reported from a sample of 526 healthy, HIV-negative subjects randomized from the general population living in an Italian north-western region (Piedmont), traditionally considered as non-endemic for leishmaniasis [7]. 

Furthermore, recently 14 cases of VL in our region (Emilia-Romagna) in a period of 6 months, from November 2012 to March 2013, were described, although in five out of these cases the diagnosis was supported by serological and clinical data alone [9]. 

**Table 2:** Demographic, epidemiological and clinical data of patients with leishmaniasis.

Patient	Origin	Sex	Age	Year of Sampling	Clinical and Epidemiological Notes	Sample type (No. of Samples)	Isoenzymes Characterization (No. of Samples)
**Visceral Leishmaniasis**							
1	IT	M	49	1994	No travel history; transplanted kidney patient; night fever	Bone marrow (1)	*L. infantum* MON 1 (1)
2	IT	M	30	1994	Frequent travels through Crete Island in the last 2 months; worsening asthenia for 20 days, fever	Bone marrow (1)	*L. infantum* MON 1 (1)
3	IT	M	39	1996–2000	No travel history; transplanted kidney patient; fever, asthenia, splenomegaly, leukopenia, thrombocytopenia, ipergammaglobulinemia	Bone marrow (5)	*L. infantum* MON 1 (4)
4	U *	F	U **	2000	Suspected lymphoma/myeloma/VL	Bone marrow (1)	n.p.
5	Albania	M	2	2005	In Italy for 1 year; chronic anemia, progressive bone marrow aplasia	Bone marrow (2) Peripheral blood (1)	n.p.
6	Macedonia	M	U **	2005	Suspected lymphoma/myeloma/VL	Bone marrow (1)	n.p.
7	IT	M	67	2009	No travel history; fever, splenomegaly	Bone marrow (1) Splenic biopsy (1)	n.p.
**Cutaneous Leishmaniasis**							
8	IT	M	65	2012	No travel history; cutaneous nodules in the legs	Skin biopsy (1)	n.p.
9	IT	M	U **	1992	Missionary in Chad; skin lesion in the right flank 2x1 cm	Skin biopsy (2)	*L. major* MON 196 (1)
10	IT	M	U **	2000	No travel history; skin lesions in the scalp	Skin lesion aspirate (1) Skin biopsy (3)	n.p.
11	Tunisia	M	29	2000	Skin lesions in the limbs	Skin biopsy (1)	*L. major* MON 25 (1)
12	Marocco	M	U **	2001	Cutaneous granuloma	Skin biopsy (1)	*L. major* MON 25 (1)
13	Tunisia	F	33	2001	Skin lesions in the limbs	Skin biopsy (1)	n.p.
14	IT	M	68	2007	No travel history; facial skin lesion	Skin biopsy (2)	n.p.
15	IT	F	49	2011	No travel history; skin lesion	Skin lesion aspirate (1)	n.p.

Note: Legend: IT: Italian; U: Unknown; n.a.: not available information; n.p.: not performed; * It was known that this patient was immigrant although the exact origin was unknown; ** It was known that these patients were adults although the exact age was unknown.

**Table 3:** Compared results of the diagnostic assays performed on the samples of patients with leishmaniasis.

Patient	Sample Type	No. of Samples	Microscopy ^a^	Culture	Real-time PCR ^b^
1	Bone marrow	1	Positive	Positive	n.p.
2	Bone marrow	1	Positive	Positive	n.p.
3	Bone marrow	5 ^c^	Positive	Positive	n.p.
			Positive	Positive	n.p.
			Positive	Positive	n.p.
			Positive	Positive	n.p.
			n.p.	Positive	n.p.
4	Bone marrow	1	n.p.	Positive	n.p.
5	Bone marrow	2 ^d^	Positive	Positive	n.p
			Negative	Negative	n.p
	Peripheral blood	1	n.p.	Negative	n.p
6	Bone marrow	1	Negative	Positive	Positive
7	Bone marrow	1 ^d^	Negative	Negative	Negative
	Splenic biopsy	1 ^d^	n.p.	Positive	Positive
8	Skin biopsy	1	Negative	Positive	Positive
9	Skin biopsy	2	Positive	Positive	n.p.
10	Skin lesion aspirate	1	n.p.	Positive	n.p.
	Skin biopsy	3	Positive	Positive	n.p.
			Negative	Positive	n.p.
			n.p.	Positive	n.p.
11	Skin biopsy	1	n.p.	Positive	n.p.
12	Skin biopsy	1	Negative	Positive	n.p.
13	Skin biopsy	1	Negative	Positive	n.p.
14	Skin biopsy	2 ^e^	n.p. n.p.	Positive Negative	Positive Positive
15	Skin biopsy	1	n.p.	Negative	Positive

Note: Legend: ^a^: microscopic examination was performed on the basis of the availability of the specimen; ^b^: real-time PCR was performed from 2005; ^c^: samples sent in a period of 5 years; ^d^: samples sent within a few days; ^e^: samples sent in a period of 9 months; n.p.: not performed.

These data suggested us to investigate the retrospective occurrence of both visceral and cutaneous leishmaniasis in our area, located in a rural region (north-western Emilia-Romagna region) of northern Italy, taking into account that either autochthonous cases or cases acquired outside our area (in Italy or abroad) may have occurred. 

The results reported in this study, based on the direct detection of the parasite in the sample by microscopic examination, culture and/or Real-time PCR, showed an infection rate of 11.2% in a period of 22 years. In particular only 15 cases of leishmaniasis were diagnosed from 1992 to 2013 demonstrating that in the province of Parma, unlike recent reports about areas close to ours (about 100 km), this disease is not frequently revealed, despite the majority (10) of the cases occurred from 2000 till now. 

The low infection rate observed in this study (15 out of 134 patients with symptoms) could be related to the presence of false negative results (due to the lack of viability of the parasites) that may occur when culture is the unique diagnostic method used as was the case of our study before 2005. However, we concluded that we likely had no false negative results because in the patients without leishmaniasis included in this study diseases other than infectious were diagnosed explaining the reported signs and symptoms. Moreover, we are confident that the results obtained since 2005 are likely due to a real low rate of *Leishmania* spp. in our area, as a very highly sensitive assay such as Real-time PCR was added to the algorithm for the diagnosis of leishmaniasis in our laboratory.

In our experience, the absence of significant differences in the sets of clinical signs and symptoms associated with patients with leishmaniasis *versus* patients not infected with *Leishmania* spp., both for VL and CL, does not allow one to provide recommendations for improving clinical diagnosis. As a matter of fact, for all the patients included in this study the query for diagnostic investigations was ordered on the basis of signs and symptoms and/or risk factors consistent with the suspicion of leishmaniasis, taking also into account that for the majority of the patients this was done to accomplish a differential diagnosis with other diseases sharing with leishmaniasis similar signs and symptoms after consulting with the medical parasitologist in the laboratory.

A noteworthy result concerns the patients for whom *Leishmania* was detected in the splenic biopsy and not in the bone marrow, the last arriving after one month from the first sample: in this case the diagnosis was performed in the primary phases of the disease, when the parasite had not yet reached the bone marrow. A special comment however regards the kidney transplanted patients included in this study. In general, the disease is emerging in immunocompromised patients undergoing bone marrow or solid organ transplantation or treatment with biologic drugs [1], with both the classical signs of VL, such as prolonged fever hepatosplenomegaly and pancytopenia, and atypical forms, such as mucosal and cutaneous forms. Transplant recipients also seem to have an increased risk of disease recurrence or reinfection. In particular, a relapse of VL should always be expected in a kidney transplant patient with a history of leishmaniasis, and such patients should be strictly monitored not only for this potential life-threatening condition, but also for the potential role of VL in graft dysfunction and loss [19].

Among the described cases, for two specimens belonging to two different patients the result of culture was negative while PCR was positive. The cultivation of the parasite, although traditionally the reference assay for the parasitological confirmation of the clinical suspicion of leishmaniasis [14,20], is dependent on the viability of the parasite and then influenced by the collection, the transport and the preservation of the sample. This could explain the two cases positive by PCR alone reported in this study. We are confident that in these two cases the results of the Real-time PCR assay were true positive due to the typical lesions observed, pathognomonic for old world leishmaniasis.

In all cases of VL, amphotericin B was administered resulting in recovery.

Interestingly, concerning the eight cases of CL, three patients were immigrants from Maghreb countries and one Italian patient reported travels through Chad, suggesting imported leishmaniasis. Furthermore, when performed, the isoenzymes characterization demonstrated the presence of *L. major*, typically distributed in northern and western Africa; in particular *L. major* MON 25 was described as the causative agent of zoonotic CL in Tunisia and in other Maghreb countries such as Morocco and Algeria [21]. In a recent study in which the analysis of isoenzymes was performed on a large number of isolates from Old World cutaneous leishmaniasis foci, *L. major* MON 196 was found in a strain from Chad [22], the same origin of the strain isolated in our study from patient No. 9. These data about the origin are not unexpected since it could be postulated that only *Leishmania infantum* was associated with the infections acquired in Italy [8].

On the contrary, the identification of *L. infantum* MON 1 in three cases of VL is an expected result since it is the most prevalent zymodeme both in human and canine leishmaniasis, representing approximately 70% of all identified strains worldwide and the 50% of the identified strains in Italy [23].

## 5. Conclusions

The figure of cases of leishmaniasis herein reported, even if not comparable to that described for Italian areas other than Parma, underlines that suitable tools are mandatory for diagnosis. However, in particular with regard to VL, the clinical suspicion was formulated later than the onset of the symptoms, allowing the worsening of the disease. The severity of a disease as VL and its documented spread in continental areas of Italy and Europe makes it necessary for the physicians of these areas to increase their attention about the concrete possibility of leishmaniasis (both VL and CL) for patients reporting signs and symptoms and/or risk factors consistent with this suspicion.
